# Dynamic Patterns of Threat-Associated Gene Expression in the Amygdala and Blood

**DOI:** 10.3389/fpsyt.2018.00778

**Published:** 2019-01-17

**Authors:** Adriana Lori, Stephanie A. Maddox, Sumeet Sharma, Raül Andero, Kerry J. Ressler, Alicia K. Smith

**Affiliations:** ^1^Department of Psychiatry and Behavioral Sciences, Emory University School of Medicine, Atlanta, GA, United States; ^2^Neurobiology of Fear Laboratory, Division of Depression and Anxiety Disorders, McLean Hospital, Belmont, MA, United States; ^3^Department of Psychiatry, Harvard Medical School, Boston, MA, United States; ^4^Institut de Neurociènces, Universitat Autònoma de Barcelona, Bellaterra, Spain; ^5^CIBERSAM, Corporació Sanitaria Parc Taulí, Sabadell, Spain; ^6^Department of Psychobiology and Methodology in Health Sciences, Universitat Autònoma de Barcelona, Bellaterra, Spain; ^7^Department of Gynecology and Obstetrics, Emory University School of Medicine, Atlanta, GA, United States

**Keywords:** threat, fear, PTSD (post-traumatic stress disorder), amygadala, stress

## Abstract

Stress and trauma profoundly influence psychiatric biobehavioral outcomes. The identification of treatment and biomarker targets would be accelerated by a broad understanding of the biological responses to these events. The goal of this study was to determine genes responsive to auditory fear conditioning (FC), a well-characterized amygdala-dependent rodent model of threat-exposure, in the presence or absence of prior stress history, providing insight into the physiological processes underlying response to trauma. RNA-sequencing was performed in blood and amygdala from mice that underwent fear conditioning with (Immo+FC) and without (FC) prior immobilization stress, a paradigm that induces HPA axis, and behavioral stress sensitization. In the amygdala, 607 genes were regulated by FC vs. home-cage (HC) controls, and 516 genes differed in stress-sensitized mice (Immo+FC vs. FC). In the former, we observed an enhancement of specific biological processes involved in learning and synaptic transmission, and in the latter processes associated with cell proliferation and the cellular response to drugs. In the blood of stress-sensitized animals, 468 genes were dynamically regulated when compared to FC, and were enriched for the biological pathways of inflammation and cytokine signaling. This study identified genes and pathways that respond to threat in the amygdala and blood of mice with and without a prior stress history and reveals the impact of stress history on subsequent inflammation. Future studies will be needed to examine the role of these dynamically regulated genes may play in human clinical stress and trauma-related disorders.

## Introduction

Post-traumatic stress disorder (PTSD) is a pervasive and debilitating psychiatric disorder that develops in vulnerable individuals after exposure to variable levels of trauma. A prior stress event, particularly early life trauma or abuse, has also been shown to cause poor psychiatric outcomes in adults, increasing risk for and the severity of PTSD (1, 2). The characteristic features of PTSD, such as hypervigilance and heightened startle reactions (DMS-V), associate with a patient's inability to regulate their fear response in the presence of a non-threatening situation (3, 4). Human neuroimaging studies and animal models have well established that the amgydala plays a central role in the processing of fearful and threatening stimuli and in mediating the constellation of responses that are associated with fear and threat-related behaviors (5, 6). Accumulating and compelling evidence now suggests that PTSD is associated with dysregulation of the amygdala, generally hyperactivity, in response to trauma-relevant or emotionally salient cues (7, 8).

Recent studies have identified differential gene expression patterns in blood between PTSD cases and trauma-exposed controls, reporting possible genes, and pathways associated with PTSD, several of which show dysregulation of the immune system and glucocorticoid pathways (9). However, there is limited knowledge of the degree of correlation between gene expression changes, accompanying trauma, and psychiatric conditions (e.g., PTSD) in the blood and those in the brain. Additionally, because of the obvious limitations of availability of human brain tissues, there had been paucity of brain-based transcriptomic studies. While transcriptomic studies from human post-mortem issues can aide in examining persistent, long-lasting changes in gene expression relevant to specific disease states, they do not permit examination of the transcriptional changes which occur in brain regions relevant for stress and trauma-related disorders at times proximal to trauma.

In this regard, traumatic memory formation in animal models can facilitate identification of genes whose expression is comparable between the amygdala and blood. As such, studies employing rodent models of stress and threat exposure may present a powerful approach toward bridging this gap. Moreover, studies examining the molecular mechanisms associated with the formation and persistence of threat-relevant memories have largely utilized Pavlovian fear-conditioning (FC), employing as conditioned stimulus either novel auditory cues to result in a largely amygdala dependent memory, or spatial, and contextual cues that integrate hippocampal and amygdala regions to regulate learning (5, 10). As much work has shown in human clinical studies has noted the importance of the amygdala in the pathophysiology of trauma-relevant disorders (11–13) and the impact of prior stress history on later risk for the development of PTSD accompanying trauma (14), we were most interested in examining the molecular changes occurring within the amygdala in the time period proximal to trauma. To meet this objective we used paradigm previously utilized by our group which has confirmed that mice immobilized for one 2 h session, 1 week prior to auditory FC have impaired fear extinction and retention, phenotypes that are seen in human clinical PTSD (15, 16). In addition to the observed behavioral phenotype, mice exposed to the immobilization paradigm had hypothalamic-pituitary-adrenal (HPA) axis hypersensitivity, and transient changes in plasma corticosterone levels (4, 17). HPA axis abnormalities, such as low levels of cortisol in urine and plasma or higher suppression of cortisol in response to dexamethasone have been also reported in PTSD patients (18, 19). As the prior (immobilization) stress history model replicates many of the behavioral and hormonal alterations that are observed with human clinical trauma-relevant disorders (20, 21), we utilized a robust mouse model of stress exposure and auditory fear conditioning to identify changes in gene expression in the amygdala response to fear conditioning with and without prior stress.

Ultimately, our goal was to identify correlated patterns of gene expression between blood and brain under these conditions to facilitate interpretation of blood-based studies of PTSD and to provide new insight into the pathophysiology stress-related disorders.

## Methods

### Animals

All experiments were performed on adult male wild-type C57BL/6J mice aged 2–3 months obtained from The Jackson Laboratory. Male mice were group-housed in a temperature-controlled vivarium with set-point maintained at 72° F (±1°) and relative humidity controlled at 40–50%, with *ad libitum* access to food and water. Each experimental group consisted of 12 mice maintained on a 12-h light/dark cycle, with all behavioral procedures being performed during the light cycle. All procedures used were approved by the Emory University Institutional Animal Care and Use Committee (IACUC) and in compliance with National Institutes of Health Guide for the Care and Use of Laboratory Animals.

### Mouse Immobilization Stress (Immo) and Fear Conditioning (FC)

Immobilization stress (Immo) and fear conditioning (FC) were conducted following the protocol from Andero et al. (4). Briefly, immobilization procedures were conducted in a room separate from housing and behavioral paradigms. Each animal was immobilized by restraining their four limbs with tape in a prone position to metal arms attached to a wooden board for 2 h. All cage-mate animals received the same treatment—either Immo or handling. Handling lasted ~1 min per mouse and consisted of letting the animal walk on top of their home cage and in the hands of the experimenter. After Immo, animals were returned to their home cage (HC) where they remained undisturbed for a week prior to fear conditioning (FC), which was performed in Immo animals and a subset of naïve animals. For auditory fear conditioning mice were habituated to white-light illuminated, standard rodent modular test chambers (ENV-008-VP; Med Associates Inc., St. Albans, VT) with an inside area of 30.5 cm (L) × 24.1 cm (W) × 21.0 cm (H) for 10 min on 2 consecutive days prior to fear conditioning. Fear conditioning consisted of five trials of a novel tone conditioned stimulus (CS; 30 s tone, 6 kHz, 70 dB), which co-terminated with a foot-shock (500 ms, 0.6 mA) unconditioned stimulus (US). The tone conditioned stimulus was generated by a Tektronix function generator audio oscillator delivered through a high-frequency speaker (Motorola, Model 948) attached to the side of each chamber. The Pre-CS period lasted 180 s and a variable inter-trial interval (ITI) was used between each CS-US pairing to result in a total conditioning session which lasted 840 s. The apparatus was cleaned with Quatricide® after each mouse.

Mice were sacrificed under basal conditions (HC group) or 2 h following auditory fear conditioning (Immo+FC and FC alone groups)—a time point that our group has consistently utilized for looking at changes in transcriptional processes in the amygdala following auditory fear conditioning—with a brief exposure to isoflurane anesthesia, <30 s, followed by decapitation and trunk blood collection. Trunk blood from two mice of the same behavioral group was collected into a single 3 ml EDTA BD-Vacutainer tubes. A 250 μL aliquot of each blood sample was allocated for complete blood count, and the remaining sample was stored at −80°C. Brains were immediately frozen on dry ice and stored at −80°C, and 1 week later brains were mounted on a sliding, freezing microtome using Tissue-Tek OTC, and sectioned slowly to approximately Bregma −1.34 mm (22) to reveal the amygdala. One millimeter of bilateral amygdala punches, centered on the basolateral nucleus were taken and immediately frozen in microcentrifuge tubes on dry ice and stored at −80 degrees for later RNA extraction. The bilateral amygdala punches (and the blood) from 2 mice of the same behavioral group were pooled together, thus resulting in a total of 6 pooled samples for each behavioral condition that were sequenced. As the murine basolateral amygdala is slightly larger than 1 mm, we cannot exclude the possibility that other amygdala subregions were including in these tissue samples.

### RNA Extraction and Sequencing

RNA extraction, QC, library preparation, and sequencing were conducted by the Yerkes Non-Human Primate Genomics Core (Atlanta, GA). Amygdala punches were homogenized with a bead milling homogenizer, and total RNA was isolated and purified from each sample with the RNeasy Mini Kit (Qiagen, CA) following the manufacturer's instructions. RNA quality and quantity were verified with the 2100 BioAnalyzer PicoChip (Agilent Technologies, Santa Clara, CA) before sequencing, and all samples had an RNA Integrity Number (RIN) score of nine or higher. For blood samples, globin mRNA transcripts were depleted using the GLOBINclear™-Mouse kit (Ambion, Austin, TX) according to the manufacturer's instructions. Briefly, 1 μg of total RNA was treated with biotinylated oligonucleotides to selectively deplete the α- and β-globin sequences. Subsequently, streptavidin paramagnetic beads were added, to capture the hybridized globin mRNA biotinylated probes, resulting in an average 25% loss of total RNA. Further purification of the globin depleted RNA was performed with SPRI magnetic beads as per manufacturer's recommendation.

Libraries were prepared using the Illumina (Illumina Inc. San Diego, CA) TruSeq™ RNA kit as per manufacturer's instructions. Briefly, 250 ng of total RNA was used for library preparation. The TruSeq method (low-throughput protocol) employs two rounds of poly-A based mRNA enrichment using oligo-dT magnetic beads followed by mRNA fragmentation using cations at high temperature. First and second strand cDNA synthesis was performed followed by end repair of the blunt cDNA ends. One single “A” base was added at the 3′ end of the cDNA followed by ligation of barcoded adapter unique to each sample. The adapter-ligated libraries were then enriched using PCR amplification. The amplified library was validated using a High Sensitivity DNA chip on the Agilent Bioanalyzer. The libraries were further quantified on Qubit® 2.0 Fluorometer (Life Technologies, Grand Island, NY) using the High Sensitivity dsDNA assay. Each library contained the same amount of RNA, and eight sample pools were multiplexed in each lane of the flowcell. PhiX was used as an internal control on each lane to monitor the error statistics, and sequencing was performed on the Illumina HiSeq1000 system employing a paired-end 101 cycles run.

### Statistical Analysis

Alignment to the 10 mm UCSC Mouse Assembly was performed using STAR version 2.3 (23); parameters were set using the annotation as a splice junction reference. Sample reads were assembled into transcript models using cufflinks (v2.1.1), which were then merged and processed with cuffdiff v2.1.1 (24) to produce per sample FPKM expression levels and estimate differential expression between the sample groups. Each tissue (amygdala and blood) was processed separately to allow for identification of tissue-specific differences for each behavioral condition. The false discovery rate (FDR) was controlled at 5% to account for multiple testing in all analyses (*q* < 0.05). Volcano plots were generated in R. Differentially expressed genes were further evaluated for the enrichment of biological processes using DAVID 6.8 (25). Differences in cell counts between groups were evaluated using an independent *t*-test.

## Results

In this study, two groups of mice (with and without a history of immobilization stress; Immo), were trained in an auditory fear conditioning paradigm (Immo+FC and FC, respectively); a third group of naïve, home-cage control animals was handled and removed from the vivarium but not exposed to any behavioral intervention (HC). All mice were sacrificed together 2 h after last fear conditioning (or an equivalent time of day after handling), and gene expression patterns from blood and amygdala were compared. Examination of the freezing behaviors of animals in the Immo-FC and FC groups did not reveal any significant differences in baseline, pre-tone CS freezing (*t* = −0.39, *p* > 0.05) or tone CS freezing across the auditory conditioning session (*t* = 0.43, *p* > 0.05). Table S1 shows the total number of expressed genes in both tissues across the three different groups (HC, FC or Immo+FC). We observed tissue-specific gene expression, with an overall higher number of genes expressed in the amygdala relative to the blood in all three groups (HC, FC, Immo+FC). Overall 11,353 genes were expressed in both tissues, with 580 uniquely expressed in blood and 4,271 uniquely expressed in the amygdala.

### Fear Conditioning Induces Robust Gene Expression Differences in Amygdala and Blood

**Figure 1:**
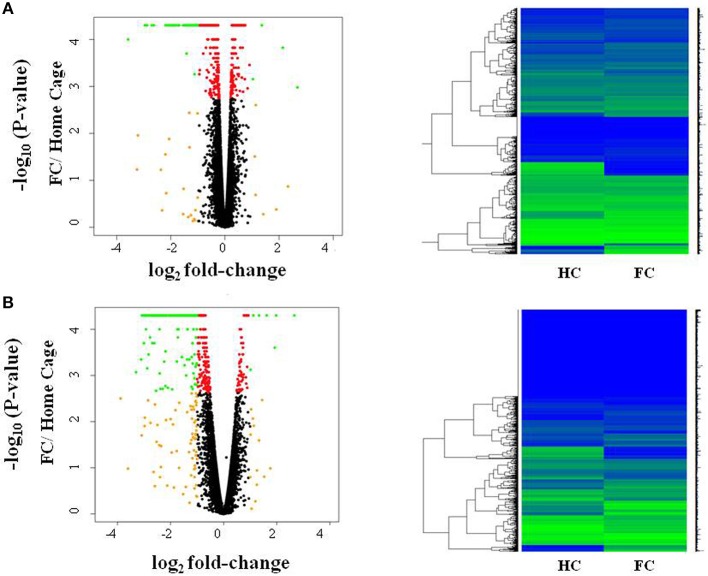
Fear conditioning induces gene expression differences in amygdala and blood. Volcano plots and heatmaps show changes in gene expression in the amygdala **(A)** and blood **(B)** after fear conditioning compared to Home Cage (HC vs. FC). The horizontal axis of the Volcano plot is log_2_ fold change for differently expressed genes, and the vertical axis is the negative-log_10_ of the *p*-values are plotted. Each dot represents a gene, with red dots showing genes reaching an FDR corrected *p*-value of 0.05, and green dots representing genes with FDR < 0.05 and absolute fold change >1; orange dots have an absolute fold change>1 but do not reach experiment-wide significance; black dots are genes whose expression is similar between the two groups.

We first investigated differences in response to fear conditioning (FC vs. HC). Figure 1A shows that FC induced gene expression changes in the amygdala, with 607 genes differentially expressed when compared to HC (Table S2; FDR < 0.05). FC resulted in a down-regulation of gene expression in the majority of these genes (76.6%). We then evaluated differentially expressed genes for enrichment of biological process and identified 15 processes that were enriched after multiple test correction (Table 1, Table S3). Among these processes, there was an enrichment of specific biological processes including memory formation and consolidation, and neurotransmission, with learning or memory (*p* = 4.18 × 10^−4^), and associative learning (*p* = 0.01).

**Table 1:** Enrichment biological process analyses for differential express genes in fear conditioning mice compared to control (FC vs. HC).

**GO number**	**Description**	**FE^**a**^**	***p*-value^**b**^**
**AMYGDALA**			
GO:0007611	Learning or memory	8.20	4.18 × 10^−4^
GO:0008306	Associative learning	8.47	0.010
GO:0007268	Chemical synaptic transmission	3.65	0.008
GO:0003007	Heart morphogenesis	5.99	0.008
GO:0035556	Intracellular signal transduction	2.44	0.012
GO:0055085	Transmembrane transport	2.49	0.023
GO:0007507	Heart development	2.80	0.025
GO:0048511	Rhythmic process	3.81	0.025
GO:0007612	Learning	5.28	0.030
GO:0007157	Heterophilic cell-cell adhesion via plasma membrane cell adhesion molecules	6.03	0.027
GO:0045944	Positive regulation of transcription from RNA polymerase II promoter	1.75	0.036
GO:0006811	Ion transport	2.03	0.036
GO:0006351	Transcription, DNA-templated	1.50	0.041
GO:0045053	Protein retention in Golgi apparatus	27.87	0.040
GO:0007626	Locomotory behavior	3.87	0.045
**BLOOD**			
GO:0007156	Homophilic cell adhesion via plasma membrane adhesion molecules	12.4	1.63 × 10^−26^
GO:0002376	Immune system process	3.07	0.009

a *FE, Fold Enrichment*.

b *p-value following Benjamini-Hochberg correction for multiple testing*.

In blood, FC results in expression differences of 352 genes in blood relative to HC (Table S4; FDR < 0.05; Figure 1B), with the majority of genes identified (84.3%) having lower expression levels in FC when compared to HC. Only two biological processes were enriched among these differentially expressed genes (Table 1; Table S3), homophilic cell adhesion of plasma membranes of adjacent cells (*p* = 1.6 × 10^−26^), and immune system processes (*p* = 0.009). Comparison of genes regulated in amygdala (FDR < 0.05) with those regulated in blood (*p* < 0.05) revealed 32 genes differentially expressed in FC vs. HC, 9 of which reached FDR significance in both tissues (Table 2).

**Table 2:** Genes differentially expressed in FC compared to HC in amygdala and blood.

**Gene**	**Amygdala**			**Blood**		
	**Fold change**	***p*-value**	***q*-value**	**Fold change**	***p*-value**	***q*-value**
***Nxpe4***	−0.39	0.0003	0.010	−1.27	5.00*E*−05	0.002
***Plxnd1***	−0.22	0.0017	0.047	−0.82	5.00*E*−05	0.002
***C1ql3***	0.24	0.0012	0.037	−2.41	0.0001	0.004
***Zhx2***	0.29	0.0012	0.037	0.68	0.0001	0.006
***Pbrm1***	−0.26	0.0001	0.005	−0.68	0.0005	0.015
***Adam8***	0.47	0.0006	0.020	0.62	0.0008	0.022
***Zbtb40***	0.37	0.0002	0.007	0.67	0.0009	0.026
***Myo5a***	−0.30	0.0001	0.003	−0.53	0.001	0.030
***Dmxl2***	−0.42	0.0001	0.003	−0.66	0.002	0.045
*Kif1b*	−0.25	0.0012	0.036	−0.53	0.003	0.054
*Sorl1*	−0.37	0.0001	0.003	0.51	0.004	0.078
*Sdc4*	0.26	0.0008	0.025	0.46	0.005	0.088
*Thada*	−0.33	0.0010	0.032	0.49	0.006	0.098
*Tra2a*	0.32	0.0001	0.003	−0.45	0.006	0.106
*Insr*	−0.22	0.0015	0.044	−0.49	0.008	0.120
*Galnt9*	0.38	0.0001	0.003	−0.58	0.009	0.137
*Myadm*	0.22	0.0013	0.039	−0.48	0.011	0.152
*Abhd2*	−0.25	0.0007	0.023	0.50	0.013	0.172
*Klf11*	−0.41	0.0009	0.028	−0.48	0.016	0.195
*Zfp280c*	−0.31	0.0001	0.005	−0.50	0.020	0.225
*Bptf*	−0.25	0.0001	0.003	0.38	0.020	0.229
*Numb*	0.26	0.0005	0.017	0.39	0.022	0.241
*Pds5a*	−0.29	0.0001	0.005	0.39	0.023	0.248
*Sdf2l1*	0.58	0.0001	0.003	0.44	0.023	0.248
*Egr3*	0.33	0.0001	0.003	0.89	0.024	0.251
*Arhgef12*	−0.24	0.0004	0.015	−0.40	0.025	0.260
*Kat6a*	−0.28	0.0001	0.003	0.36	0.030	0.288
*Guf1*	−0.27	0.0006	0.021	−0.45	0.036	0.320
*Phka2*	−0.47	0.0001	0.003	−0.42	0.040	0.344
*Zfp445*	−0.21	0.0016	0.046	−0.33	0.044	0.362
*Dusp1*	−0.54	0.0001	0.003	0.37	0.045	0.364
*Fkbp5*	0.24	0.0007	0.024	0.35	0.048	0.377

*Bold Gene Names: Adam8, a disintegrin and metallopeptidase domain 8; C1ql3, C1q-like 3; Dmxl2, Dmx-like 2; Myo5a, myosin VA; Nxpe4, Neurexophilin and PC-Esterase Domain Family, Member 4; Pbrm1, Polybromo 1; Plxnd1, Plexin D1; Zbtb40, Zinc finger and BTB domain containing 40; Zhx2, Zinc fingers and homeoboxes 2. ^b^C1ql3 (C1q-like 3) changes in different direction in brain and blood*.

### Prior Stress Sensitization Induces Gene Expression Changes in Response to Fear Conditioning

**Figure 2:**
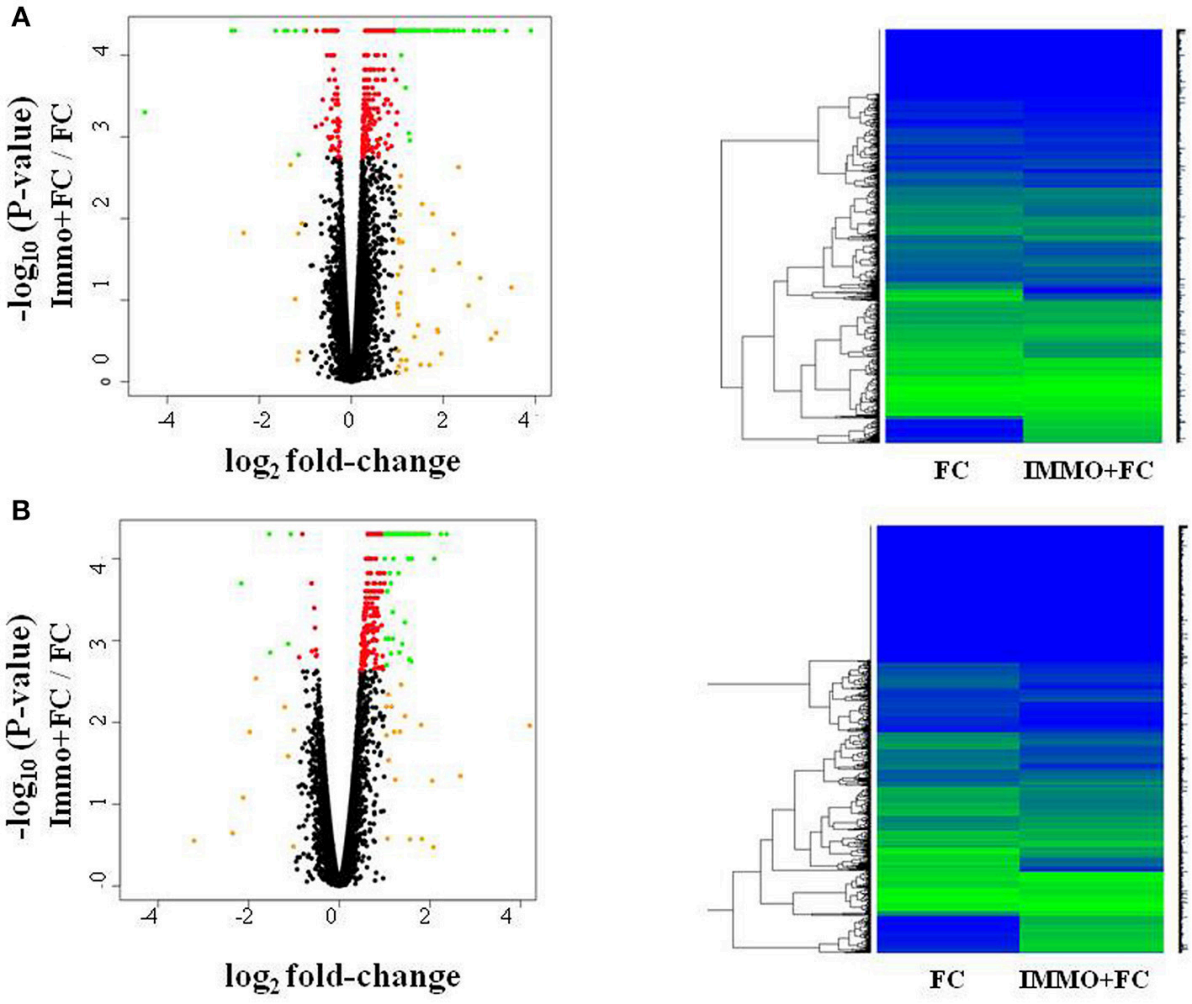
Fear conditioning and prior immunization induce gene expression differences in amygdala and blood. Volcano plots and heatmaps show changes in gene expression in the amygdala **(A)** and blood **(B)** of animals that experienced immobilization (Immo+FC) prior to fear conditioning (FC). The horizontal axis of the Volcano plot is log_2_ fold change for differently expressed genes, and the vertical axis is the negative-log_10_ of the *p*-values are plotted. Each dot represents a gene, with red dots showing genes reaching an FDR corrected *p*-value of 0.05, and green dots representing genes with FDR < 0.05 and absolute fold change >1; orange dots have an absolute fold change>1 but do not reach experiment-wide significance; black dots are genes whose expression is similar between the two groups.

**Figure 3:**
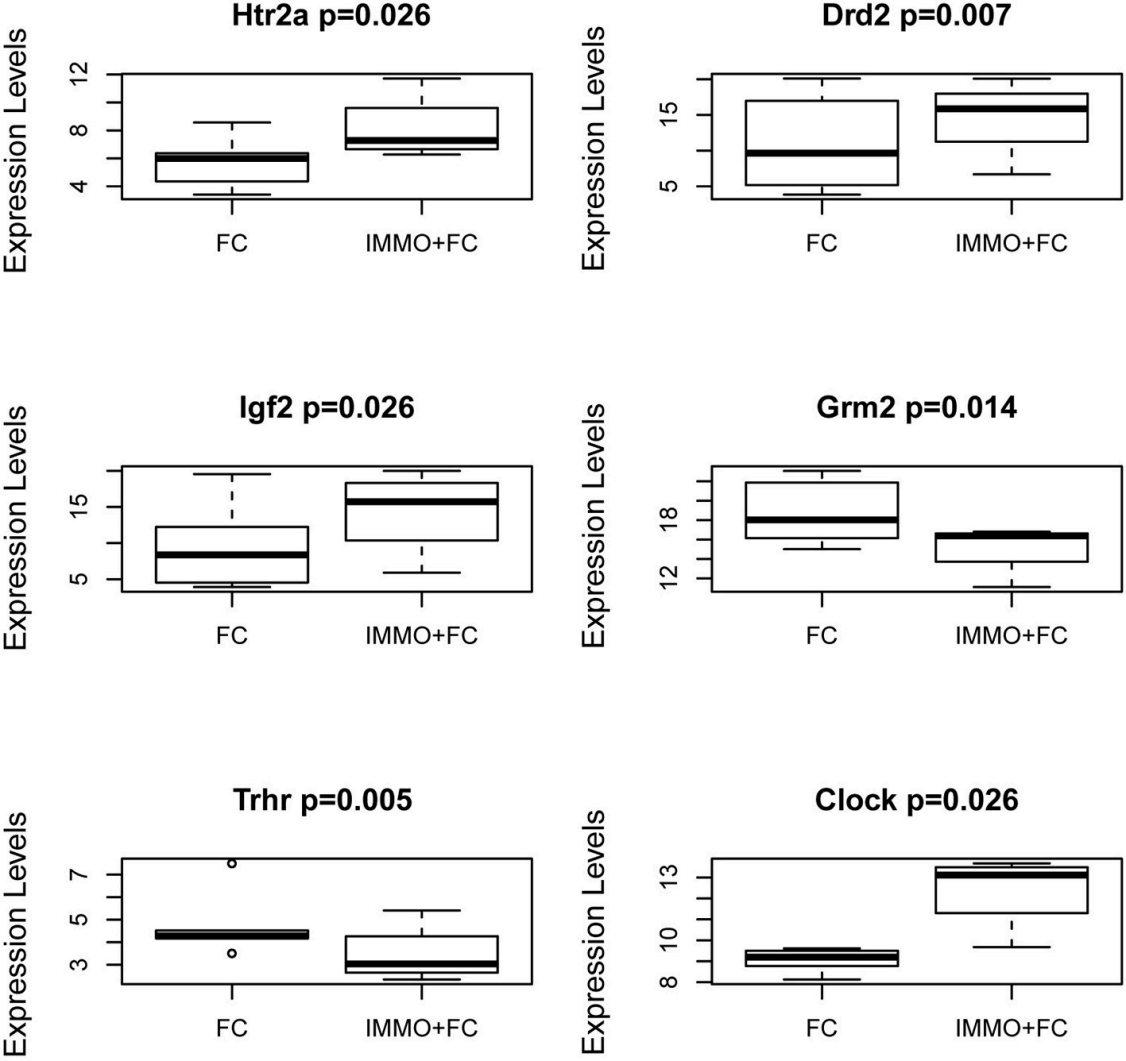
Genes in the Amygdala traditionally associated with PTSD or psychiatric stress phenotypes. Box and Whiskers plots of gene expression in FC and Immo+FC animals for (a) 2 genes traditionally associated with PTSD (*Htr2a* and *Drd2*), and (b) 4 genes associated with stress phenotypes (*Igf2, Grm2, Trhr*, and *Clock*). On the y-axis, gene expression values from the different animal groups. The box represents their quartiles; whiskers the variability outside the upper and lower quartiles; the horizontal line represents the median.

To evaluate how prior stress exposure may alter the transcriptional processes that accompany fear conditioning, we compared amygdala expression in the Immo+FC to the FC group (Figure 2A). We identified 516 genes that had differences in expression levels (FDR < 0.05; Table S5, Figure 2A), 84.3% of which were higher in FC. Among those, we observed genes that have been associated with PTSD in previous human studies (e.g., *DRD2* and *HTR2a*) (26–29) or linked to anxiety or PTSD-like behaviors in humans or animal models (*Igf2, Grm2, Clock, Trhr*) (30–32) (Table S5; Figure 3). Enrichment analyses of the 516 differentially expressed genes revealed four biological pathways, including cell proliferation and cellular response to drugs (Table 3; Table S6).

**Table 3:** Enrichment of biological process among genes associated with prior stress immobilization.

**GO number**	**Description**	**FE^**a**^**	***p*-value^**b**^**
**AMYGDALA**			
GO:0008285	Negative regulation of cell proliferation	3.43	0.004
GO:0006457	Protein folding	5.61	0.010
GO:0006986	Response to unfolded protein	9.39	0.014
GO:0042493	Response to drug	3.18	0.029
**BLOOD**			
GO:0002376	Immune system process	6.51	2.29 × 10^−25^
GO:0045087	Innate immune response	6.01	6.40 × 10^−23^
GO:0006954	Inflammatory response	5.83	3.38 × 10^−18^
GO:0052697	Xenobiotic glucuronidation	39.59	5.18 × 10^−8^
GO:0032496	Response to lipopolysaccharide	4.97	1.61 × 10^−6^
GO:0030593	Neutrophil chemotaxis	8.39	1.40 × 10^−5^
GO:0006955	Immune response	3.93	1.72 × 10^−5^
GO:0006935	Chemotaxis	6.04	1.70 × 10^−5^
GO:0042742	Defense response to bacterium	4.66	1.54 × 10^−5^
GO:0032760	Positive regulation of tumor necrosis factor production	9.06	1.47 × 10^−5^
GO:0009813	Flavonoid biosynthetic process	16.97	4.82 × 10^−5^
GO:0052696	Flavonoid glucuronidation	16.97	4.82 × 10^−5^
GO:0071223	Cellular response to lipoteichoic acid	29.69	1.19 × 10^−4^
GO:0009615	Response to virus	6.36	4.56 × 10^−4^
GO:0050729	Positive regulation of inflammatory response	7.07	0.0017
GO:0032755	Positive regulation of interleukin-6 production	7.71	0.0028
GO:0002755	MyD88-dependent toll-like receptor signaling pathway	16.70	0.0028
GO:0019221	Cytokine-mediated signaling pathway	4.27	0.0033
GO:0055072	Iron ion homeostasis	8.48	0.0045
GO:0050830	Defense response to Gram-positive bacterium	5.27	0.0051
GO:0031663	Lipopolysaccharide-mediated signaling pathway	10.06	0.0062
GO:0042127	Regulation of cell proliferation	3.34	0.006
GO:0045766	Positive regulation of angiogenesis	4.42	0.009
GO:0050873	Brown fat cell differentiation	9.17	0.009
GO:0010628	Positive regulation of gene expression	2.57	0.009
GO:0050728	Negative regulation of inflammatory response	5.12	0.013
GO:0042535	Positive regulation of tumor necrosis factor biosynthetic process	17.13	0.013
GO:0002548	Monocyte chemotaxis	7.79	0.020
GO:0034341	Response to interferon-gamma	10.28	0.019
GO:0071222	Cellular response to lipopolysaccharide	3.20	0.021
GO:0008360	Regulation of cell shape	3.84	0.022
GO:0007165	Signal transduction	1.70	0.026
GO:0006898	Receptor-mediated endocytosis	5.94	0.026
GO:0051607	Defense response to virus	3.47	0.026
GO:0043123	Positive regulation of I-kappaB kinase/NF-kappaB signaling	3.59	0.035
GO:0032494	Response to peptidoglycan	22.27	0.036
GO:0050766	Positive regulation of phagocytosis	6.49	0.041
GO:0035456	Response to interferon-beta	19.79	0.050
GO:0045410	Positive regulation of interleukin-6 biosynthetic process	19.79	0.050

a *FE, Fold Enrichment*.

b *p-value following Benjamini-Hochberg correction for multiple testing*.

**Figure 4:**
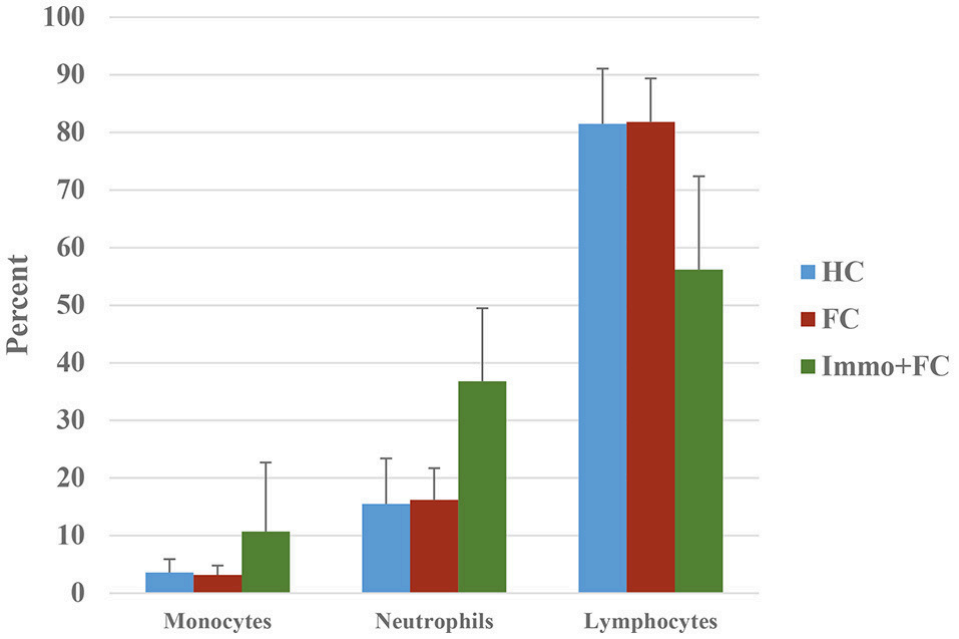
Prior stress sensitization causes immune dysregulation. Percent of monocytes, neutrophils, and lymphocytes in the three different groups (HC, FC, and Immo+FC) are shown. Bars indicate standard errors.

In blood, prior stress history (Immo+FC) associates with expression differences in 468 genes relative to FC (Figure 2B), the majority of which (97%) increased in expression compared to FC (FDR < 0.05; Table S7). Enrichment analysis revealed 39 pathways including immune response, inflammation, and cytokine signaling pathways (Table 3). To contextualize these differences, we compared the proportion of blood cell types (monocytes, neutrophils, and lymphocytes) between each group. Although there were no differences in blood cell composition between the HC and FC groups, Immo+FC had a higher proportion of neutrophils and a lower proportion of lymphocytes relative to both FC (*p* = 0.013) and HC (*p* = 0.007; Figure 4).

**Figure 5:**
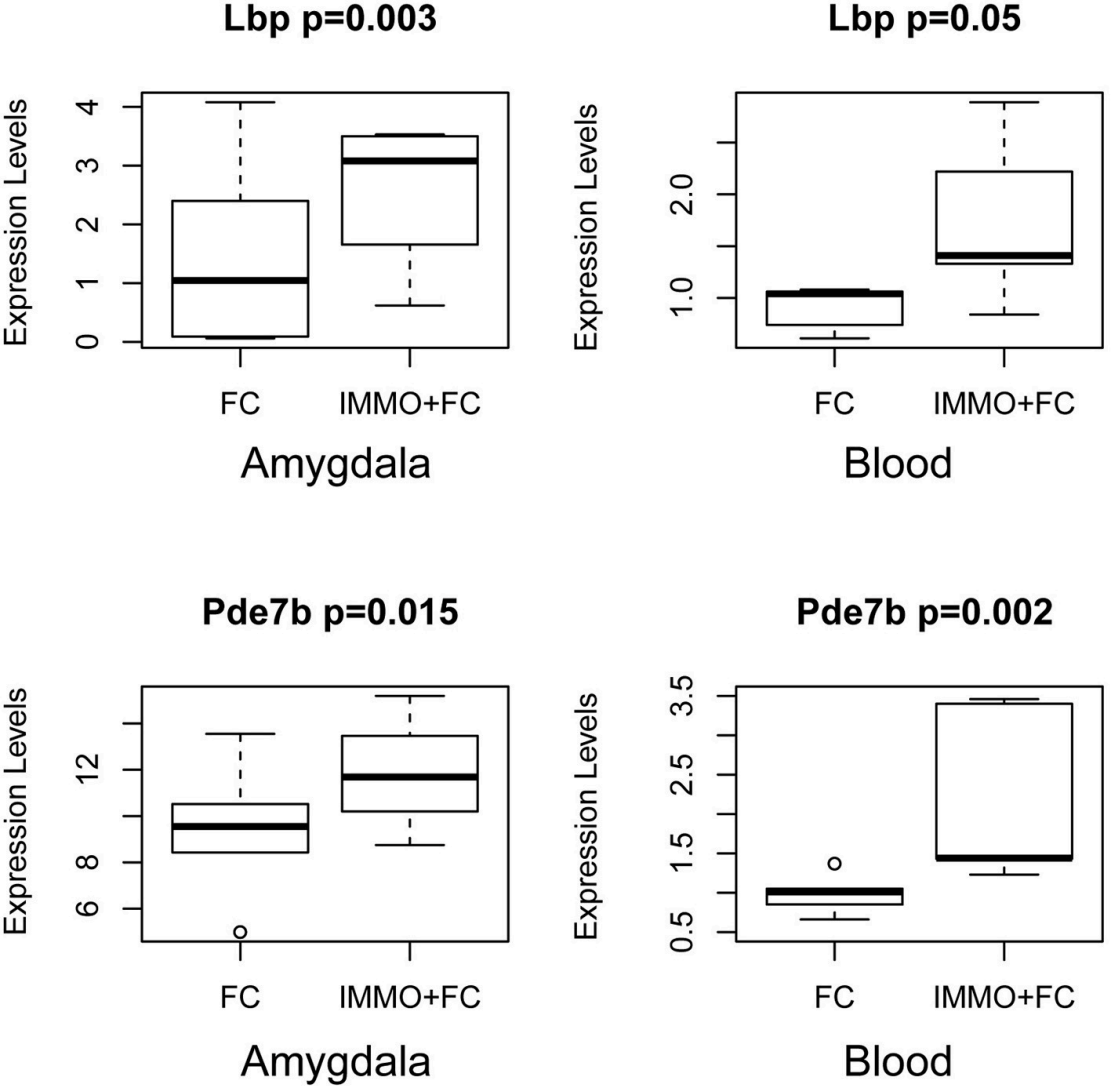
Common Signals in Amygdala and Blood prior stress sensitization in representative genes. Box and Whiskers plots depict gene expression (y-axis) in FC and Immo+FC animals. Representative genes that associate with prior immobilization in both amygdala and blood are shown. On the y axis, gene expression values from the different animal group. The box represents their quartiles; whiskers the variability outside the upper, and lower quartiles; the horizontal line represents the median; outliers are indicated by single dots.

We then compared the genes whose expression differed in the amygdala (FDR < 0.05) with those that differed in blood. We identified 27 genes (Table 4) that change in Immo+FC vs. FC in both tissues, 20 (74%) of which occurred in the same direction in both tissues. Among the 10 genes that remained significant after multiple test correction in both tissues, *Dmxl2, Trps1, Fgd4*, and *Thbd* have similar expression patterns in Immo+FC and HC. Interestingly, the remaining genes in which immobilization (Immo+FC) had induced changes in expression seem to be involved in immune response (*Lbp* and *Lnc2*), anxiety behavior and schizophrenia (*Pde7b*), corticosterone homeostasis, and steroid transportation (*Lcn2* and *Soat1*; Figure 5). While we cannot state whether or not these genes are also regulated by immobilization alone, the observation of regulation in both stress exposed and non exposed animals following fear conditioning suggests that these genes are similarly transcribed in the amygdala following fear conditioning.

**Table 4:** Overlap in amygdala and blood in Immo+FC vs. FC.

**Gene**	**Amygdala**			**Blood**		
	**Fold change**	***p*-value**	***q*-value**	**Fold change**	***p*-value**	***q*-value**
*Dmxl2*	0.39	5E-05	0.003	0.99	5E-05	0.002
*Erdr1*	−0.59	5E-05	0.003	−1.07	5E-05	0.002
*Lcn2*	3.36	5E-05	0.003	0.95	5E-05	0.002
*Pde7b*	0.33	4E-04	0.015	1.15	5E-05	0.002
*Trps1*	0.40	2E-04	0.007	0.81	5E-05	0.002
*Sdf2l1*	0.39	7E-04	0.024	0.64	2E-04	0.007
*Soat1*	0.34	5E-04	0.019	0.55	5E-04	0.015
*Thbd*	0.69	5E-05	0.003	0.69	7E-04	0.019
*Fgd4*	0.78	5E-05	0.003	0.84	8E-04	0.021
*Lbp*	0.83	5E-05	0.003	0.94	2E-03	0.047
*Gh*	−4.49	5E-04	0.019	−1.84	3E-03	0.058
*Cers6*	0.44	5E-05	0.003	0.55	4E-03	0.072
*Rbm3*	−0.30	1E-03	0.032	−0.46	4E-03	0.075
*Stxbp6*	−0.27	8E-04	0.025	0.74	7E-03	0.107
*Lyst*	0.53	5E-05	0.003	0.42	7E-03	0.110
*Jhdm1d*	0.35	5E-05	0.003	0.40	1E-02	0.154
*Lnpep*	3.10	5E-05	0.003	0.41	1E-02	0.178
*Endou*	1.01	5E-05	0.003	−0.76	2E-02	0.212
*Cdk5rap2*	0.72	5E-05	0.003	−0.44	2E-02	0.242
*Polr2a*	−0.26	7E-04	0.023	−0.36	3E-02	0.270
*F5*	0.60	3E-04	0.012	−0.38	3E-02	0.282
*Prkdc*	0.29	1E-03	0.031	−0.37	3E-02	0.310
*Hpcal1*	−0.31	5E-05	0.003	0.33	3E-02	0.311
*Nr4a1*	0.31	1E-04	0.005	−0.33	3E-02	0.312
*Dnajb14*	1.55	5E-05	0.003	0.54	4E-02	0.342
*Pcnx*	0.26	5E-04	0.017	0.33	5E-02	0.368
*Pisd_ps3*	−0.40	5E-05	0.003	−0.31	5E-02	0.373

*Gene associated in both blood and amygdala. Gene Name: Dmxl2, Dmx-like 2; Erdr1, erythroid differentiation regulator 1; Fgd4, FYVE, RhoGEF, and PH domain containing 4; Lcn2, lipocalin 2; Lbp, lipopolysaccharide binding protein; Pde7b, phosphodiesterase 7B; Soat1, sterol O-acyltransferase 1; Sdf2l1, stromal cell-derived factor 2-like 1; Thbd, thrombomodulin; Trps1, trichorhinophalangeal syndrome I*.

## Discussion

This study utilized a translational animal model to examine molecular alterations associated with threat exposure in both the blood and amygdala of mice with and without a prior stress history. As much work has noted that, while trauma-related disorders can occur in individuals following exposure to a single trauma, individuals with a history of early stress and trauma are more likely to develop PTSD following subsequent trauma exposure (2, 33). We examined RNA expression changes in two different tissues: (1) the amygdala, often considered the “hub” of the fear and threat response in humans and animals (34, 35) and (2) the blood, the most common tissue used in human PTSD studies. Identification of a common gene expression response pattern presents a valuable step in translational biology, toward bridging the disconnect between how peripheral gene expression changes are relevant to PTSD-related behavioral alterations in humans.

In this study, fear conditioning resulted in differences in enrichment of genes implicated in learning and memory as well as general cellular processes in the amygdala (Table 1). These results provide confidence in our approach, as they are consistent with established pathways relevant to amygdala-mediated fear learning. However, our observation of fear conditioning induced down-regulation of gene transcription in the amygdala is noteworthy as one might expect that associative learning would increase transcription to support plasticity necessary for memory formation. As fear conditioning has been found to result in multiple waves of transcriptional processes occurring across the minutes to hours that follow (36), these data should be viewed as a static snapshot of the dynamic transcriptional processes that accompany fear conditioning, i.e., 2 h for the current study. Other recent studies have utilized time-points more proximal (30 min or 1 h) to fear conditioning and more distant (6 h and 24 h) to examine transcriptional changes, and have also demonstrated conditioning related down-regulation and up-regulation of gene targets (10, 37–39). Therefore, while 2 h has traditionally been used by our group for examining transcriptional processes following fear conditioning in the amygdala, these data must indeed be considered as only a subset of the dynamic and highly interwoven molecular processes, including translational and epigenetic processes, which also accompany fear conditioning.

In response to fear conditioning, prior acute stress immobilization conducted the previous week, induces distinct gene expression differences involved in immune activation pathways in blood. Several studies have shown a strict causal association between immune response, inflammation, and PTSD (40). Compared to mice that underwent FC alone, mice exposed to prior immobilization showed gene expression patterns consistent with immune dysregulation. This was supported by the observation that blood from Immo+FC mice had a higher proportion of neutrophils, an essential part of the innate immune system, and an indicator of inflammation. The neutrophil-lymphocyte ratio has recently been used as an indicator of chronic low-grade inflammation, and known to associate with clinical outcomes in neuropsychiatric disorders (41, 42). Our data suggested that immune response genes and pathways are responsive to prior immobilization stress both in the blood and amygdala.

In examining transcriptional differences in the amygdala of mice with and without prior stress exposure, we observed a number of genes that have previously been associated with PTSD. In particular, *DRD2*, and *HTR2a* are of interest as both the dopaminergic and serotonergic systems have been traditionally implicated in the pathophysiology of PTSD (28, 43, 44). Indeed, dopamine dysregulation has been implicated in various PTSD symptoms (e.g., attention, vigilance, arousal, sleep), and *DRD2* has been associated with PTSD diagnosis (26, 29). Similarly, as serotonin and norepinephrine reuptake inhibitors (SSRI and SNRI) remain the first line pharmacotherapy for PTSD, and numerous candidate gene studies have identified a link between variants in serotonin related genes, including *HTR2a* (27, 28), our observation of differential expression of *HTR2a* is consistent with the previous literature and support for this neurotransmitter system in the consequences of threat and trauma exposure. We also identified genes (*Igf2, Clock, Grm2, Trhr1*) that have been previously associated with stress-related phenotypes. Interestingly, *Igf2* methylation has been found to associate with PTSD (45), fear extinction (30), and more classically in chronic stress response (46). Among other differentially expressed genes in Immo+FC vs. FC, *Clock*, a gene involved in the circadian rhythms, is particularly interesting as mice with mutations of this gene have altered anxiety behaviors (31), and as sleep disturbances are commonly reported by PTSD patients (47). Similarly, *Grm2* (metatropic glutamate 2 receptor) has been associated with anxiety-like behaviors in several rodent models, and activation of these receptors in the amygdala has been found to be necessary for fear related behaviors. Highly selective mGluR2/3 agonists depress excitatory neurotransmission in the amygdala (48), suggesting that such agonists may be potentially therapeutic for PTSD patients by reducing amygdala hyperactivity (32, 49). Finally, we observed regulation of the Thyrotropin Releasing Hormone Receptor1 (*Trhr1*) in Immo+FC animals; a novel finding that is of interest given its role in the hypothalamic-pituitary-thyroid axis and the observation that clinical dysfunction of the thyroid hormone system can manifest with anxiety behaviors (50). These data support the utility of this approach in examining blood and brain-based alterations in threat exposure occurring with and without a prior stress history.

As one of our main objectives was to identify common biological responses to fear and stress in both brain and blood, we evaluated shared genes in amygdala and blood. Although there was a limited overlap in individual genes that responded to each condition (Table 4), we found six genes in which changes of expression were specifically associated with immobilization. Among these genes, *Pde7* encodes phosphodiesterase-7, known for its regulation of T-cell function and association with regulation of immune response, and its inhibitors may help patients with immunological and neuro-inflammatory disorders (51). In rodents, inhibition of Pde7 regulates anxiety behaviors, mediated by increasing levels of hypothalamic thyrotropin-releasing hormone (52). Notably, a dual Pde7 and GSK-3β inhibitor significantly improves episodic and spatial memory and enhances fear memory, as well as facilitating paired-pulse inhibition and latent inhibition, both behaviors that have been found to be impaired in psychosis, suggesting that inhibition of Pde7 and GSK-3β enhances cognition (53). Lipopolysaccharide Binding Protein (*Lbp)* and Lipocalin 2 (*Lnc2)* are involved in an acute phase of immunological response and metabolic inflammation (54). Sterol O-Acyltransferase 1 (*Soat1*) plays an important role in cholesterol homeostasis regulation and metabolism, and it has been extensively studied as a target for hypercholesterolemia and Alzheimer's disease (55). The observation of immune response and inflammation-related genes is of particular interest given the recent emerging appreciation of the role of immune-response and inflammatory pathways in psychiatric disorders, including depression, autism, and trauma-related disorders (56, 57). Recent work examining the consequences of early-life inflammation via lipopolysaccharide administration has revealed impairments in fear memory extinction during adulthood (58), in line with our observation that a prior history of stress results alterations in inflammation, might suggest that prolonged inflammatory responses contribute to impaired fear extinction. Further, it is of interest that inhibition of pro-inflammatory cytokines has been suggested to facilitate fear memory extinction (59). Taken together, these findings suggest that a closer examination of the induction of inflammation and cytokine pathways in stress and trauma responses may yield new strategies for alleviating the deleterious consequences of stress and trauma.

While our data reveal transcriptional alterations in the amygdala and peripheral blood that accompany fear conditioning with and without prior stress history, it is important to note that other recent studies employing alternative models of rodent stress have examined gene expression in blood and brain (38, 60, 61). Our use of immobilization restraint stress is predicated on our prior experience with this paradigm resulting in altered fear memory processes, anxiety behaviors and HPA axis function (4, 15); as our primary goal was to examine the consequences of stress history on subsequent molecular alterations that accompany fear conditioning, we utilized this model to conduct this genetic discovery study. Importantly, while recent studies employing different stress procedures, 21-day variable stress, chronic restraint stress, repeated shock administration, and social defeat stress have been used to examine transcriptional processes in the amygdala, this single-session immobilization stress procedure is relatively acute in comparison (38, 61, 62). Coupled with our previous demonstration of altered fear and anxiety processes using this paradigm, the altered transcriptional processes observed in this current study suggest that even relatively brief exposures to stress prior to a threating situation, such as auditory fear conditioning, can be useful in revealing the lasting imprint of stress history on molecular events associated with traumatic memory formation. While it would be unwise to intensively interpret our data with relevance to those which have utilized different paradigms, it is important to highlight that many of these studies have also revealed alterations of genes that are associated with immune response, dopamine function, and glucocorticoid related signaling (38, 60, 61), which suggest that shared pathways altered by stress are emerging across a variety of behavioral models.

We acknowledge that this study has some limitations. First, although blood cell counts varied in the different groups, we were not able to account for such variation in the analysis but were able to capture the information related to that variation. Alternatively, as white blood cell counts change following immobilization, covarying for cell composition may obscure biological differences that accompany gene expression changes. Second, we focused our analyses on the amygdala, because of its role in fear and threat response behaviors. As such, we utilized auditory fear conditioning as it is well established to rely on amygdala function. As a result, we cannot extrapolate these data to other brain regions that contribute to stress and traumatic memory (e.g., hippocampus, insula, cingulate, and prefrontal cortex). Additionally, this study was conducted exclusively in male rodents, which showed a higher response to shock-induced contextual fear conditioning than females in terms of behavioral phenotypes (freezing and rearing activity) (63). However, given the higher prevalence and heritability of PTSD in females in human population (64, 65), and the emerging role of circulating estrogen levels in relation to fear and anxiety behaviors, additional studies should specifically examine transcriptional changes in females with full regard to the estrus phase. Next, in order to get sufficient RNA for a comprehensive survey of the transcriptome, we pooled two animals per sample for each group. Though this does not limit our ability to identify overall similarities and differences in different tissues of the same animals in response to threat exposure or to interpret pathway analysis, caution should be taken in interpreting the results of individual genes. Finally, we are aware that different strains of mice may generate different patterns of expression in response to FC; a cumulative analysis in different strains of mice could eventually increase the power to detect additional genes relevant to PTSD-related phenotypes (66).

In conclusion, this study provides a translational framework for mouse and human studies aimed at examining the molecular correlates that inform studies of psychiatric disorders. We were able to identify, in the amygdala, several genes that specifically respond to the stress immobilization paradigm, some of which have been traditionally associated with PTSD, and anxiety disorders. Future studies will be needed to evaluate if these genes associate with PTSD or stress-related traits in human clinical studies.

## Author Contributions

AL performed statistical analyses and contributed to writing the manuscript. SM performed the mouse experiments and contributed to study design, and manuscript writing. SS and RA helped with the mouse experiments and manuscript editing. KR helped to design the experiment, interpret the data, and write the manuscript. AS helped to design the experiment, analyze and interpret the data, and write the manuscript.

### Conflict of Interest Statement

The authors declare that the research was conducted in the absence of any commercial or financial relationships that could be construed as a potential conflict of interest.
