# Supplementary material for: Dynamic Patterns of Threat-Associated Gene Expression in the Amygdala and Blood
Source: Front Psychiatry. 2019 Jan 17;9:778. doi: 10.3389/fpsyt.2018.00778 (PMC6344436; doi:10.3389/fpsyt.2018.00778)
Supplement: Supplementary file 1 [file Table_1.DOCX]

**Table S1. Expressed Genes in Amygdala and Blood for each condition.**

|  | # Genes | %^a^ |
| --- | --- | --- |
| **Amygdala** |  |  |
| HC | 16,012 | 68.7 |
| FC | 16,055 | 68.9 |
| Immo+FC | 16,282 | 69.9 |
| Expressed in all | 15,626 | 67.1 |
|  |  |  |
| **Blood** |  |  |
| HC | 12,994 | 55.8 |
| FC | 12,795 | 54.9 |
| Immo+FC | 12,747 | 54.7 |
| Expressed in all | 11,933 | 51.2 |
|  |  |  |
| **Overlap** |  |  |
| HC | 12,418 | 53.3 |
| FC | 12,158 | 52.2 |
| Immo+FC | 12,165 | 52.2 |
| Expressed in all | 11,353 | 48.7 |
|  |  |  |

^a^Percent was calculated out of the total number of genes analyzed (23,303).
